# Pachydermoperiostosis (Touraine–Solente–Gole syndrome): a case report

**DOI:** 10.1186/s13256-018-1961-z

**Published:** 2019-02-21

**Authors:** Amir Joshi, Gaurav Nepal, Yow Ka Shing, Hari Prasad Panthi, Suman Baral

**Affiliations:** 1 Tribhuvan University Institute of Medicine, Maharajgunj, Kathmandu, Nepal; 2 National University of Singapore, 1E Kent Ridge Road, Singapore

**Keywords:** Pachydermoperiostosis, primary hypertrophic osteoarthropathy, Touraine–Solente–Gole syndrome

## Abstract

**Background:**

Pachydermoperiostosis (PDP) is a rare disorder characterized by clubbing of the fingers, thickening of the skin (pachyderma), and excessive sweating (hyperhidrosis). It typically appears during childhood or adolescence, often around the time of puberty, and progresses slowly. Clinical presentations of PDP can be confused with secondary hypertrophic osteoarthropathy, psoriatic arthritis, rheumatoid arthritis, thyroid acropachy, and acromegaly.

**Case presentation:**

A Mongolian male, aged 19 years, resident of a hilly district of Nepal, with history of consanguinity, presented to our outpatient department with chief complaints of pain and swelling in both hands and feet for 6 years. The pain was insidious in onset, throbbing in nature, and not relieved by over-the-counter medications. The patient also complained of profuse sweating, progressive enlargement of hands and feet, and gradual coarsening of facial features. On examination there were marked skin folds in the forehead, face, and eyelids. Clubbing and swelling of bilateral knee joints and ankle joints was also evident. He was subsequently investigated extensively for acromegaly. Insulin-like growth factor-1 level and oral glucose tolerance test were normal. Radiography of various bones showed periosteal hypertrophy with subperiosteal bone formation.

**Conclusions:**

PDP should be considered as a differential diagnosis when a patient presents with hypertrophic osteoarthropathy and acromegalic features.

## Background

Pachydermoperiostosis (PDP) is a rare disorder characterized by clubbing (acropachy) of the fingers and toes; thickening of the skin (pachyderma), usually of the face; excessive sweating (hyperhidrosis); and new bone formation associated with joint pain [1, 2]. In 1935, the three dermatologists Touraine, Solente, and Gole recognized this condition as a familial disorder presenting in three forms, namely complete (periostosis and pachyderma), incomplete (without pachyderma), and the forme fruste (pachydermia with minimal skeletal changes) [3]. PDP is also therefore known as the Touraine–Solente–Gole syndrome. Borochowitz *et al.* [4] previously established that the diagnosis should only be made when at least two of the following are present: positive family history, clubbing, hypertrophic skin changes, and bone pain/radiographic changes. According to Jajic et al. [5], the estimated prevalence is of approximately 0.16%. It usually manifests in adolescence, occurring almost exclusively in males, with a male to female ratio of 7:1 [6].

The pathogenesis of PDP is not fully known. The 15-hydroxyprostaglandin dehydrogenase gene and the solute carrier organic anion transporter family member 2A1 have been found to be associated with PDP [7–10]. It is thought that increased levels of prostaglandin E2 (PGE2) as a result of defective selective uptake across the plasma membrane by solute carrier organic anion transporter family member 2A1 and/or intracellular degradation by 15-hydroxyprostaglandin dehydrogenase is central to the pathogenesis of PDP [11–13]. Sasaki *et al.* [10] previously reported that pachydermia severity and the associated histological changes are correlated with serum PGE2 levels. Elevated PGE2 levels are hypothesized to induce cytokine-mediated tissue remodeling and vascular stimulation, leading to hyperhidrosis, acro-osteolysis, periostosis, arthritis, and pachyderma as seen in PDP patients [14].

Herein, we report a case of young male with chief complaints of pain and progressive enlargement of hands and feet along with profuse sweating and gradual coarsening of facial features. He was extensively investigated for acromegaly, and the final diagnosis was pachydermoperiostosis.

## Case presentation

A Mongolian male, aged 19 years, resident of a hilly district of Nepal, presented to our outpatient department with chief complaints of pain and swelling in both hands and feet for 6 years. The pain was insidious in onset, throbbing in nature and not relieved by over-the-counter medications. The patient also complained of profuse sweating, progressive enlargement of hands and feet, and gradual coarsening of facial features. His family history was significant for consanguinity – his grandparents have a consanguineous relationship. There was otherwise no history of a similar illness in the family members, and this was the first time the patient sought medical attention for this issue. There was no history of scalp dandruff or rashes, and the patient denied having symptoms such as fatigue, eye redness, eye or mouth dryness, chest pain, or exertional dyspnea. There was no history of fever, palpitations, heat intolerance, or tremors.

**Fig. 1:**
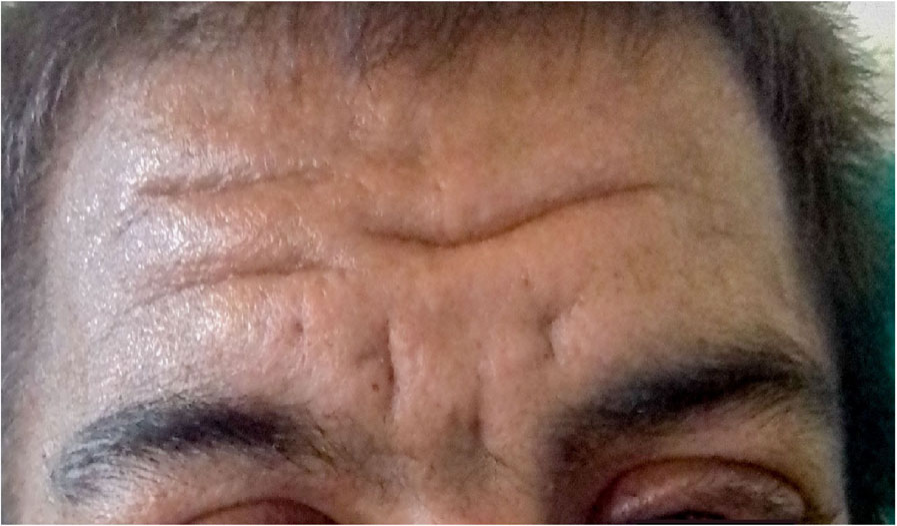
Pachyderma

**Fig. 2:**
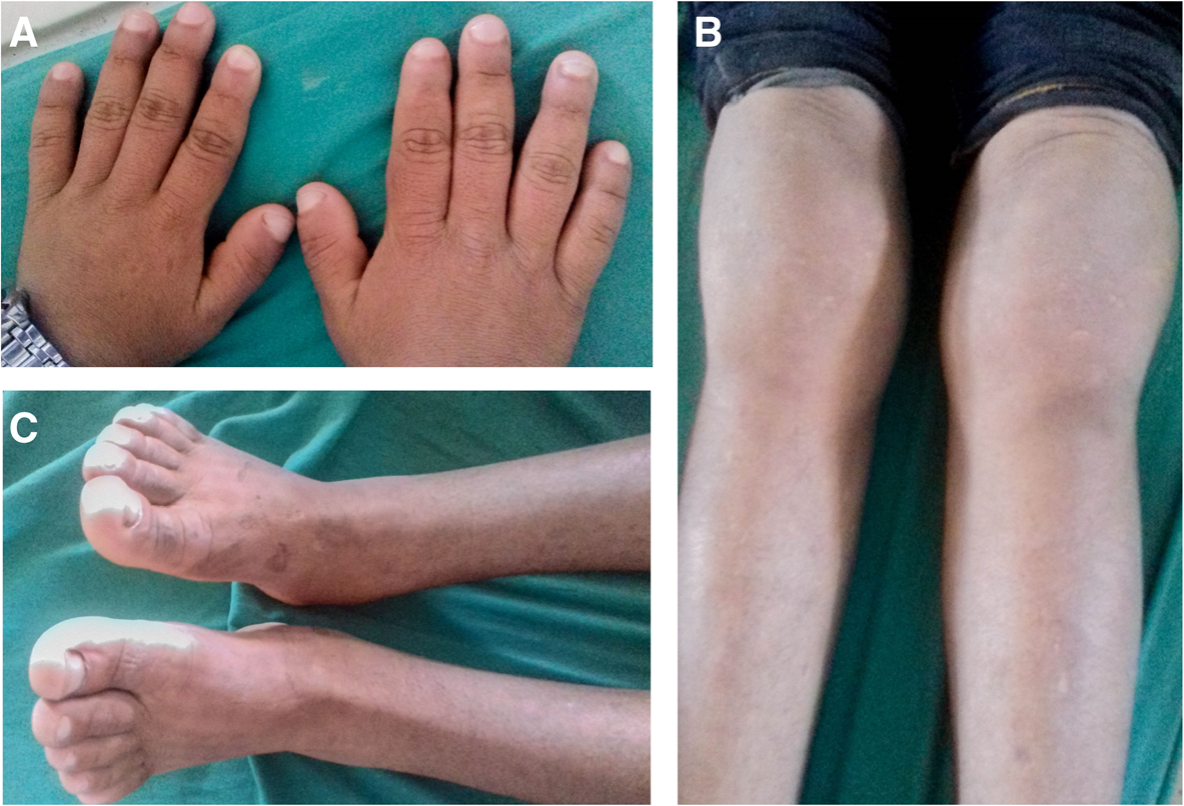
**a** Clubbing of finger nails, **b** swelling of bilateral knees, and **c** swelling of bilateral ankle joints

The patient was hemodynamically stable, alert, and conversant when he presented. On examination, there were marked skin folds in his forehead, face, and eyelids (Fig. 1). Clubbing and swelling of bilateral knee joints and ankle joints were also evident (Fig. 2). Cardiovascular, respiratory, neurological, and thyroid examination performed for the patient was otherwise unremarkable. There was no scalp dandruff, rashes, psoriatic nail changes, subcutaneous nodules, or eye redness noted on examination.

We performed biochemical investigations including a full blood count (total lymphocyte count 9.5 × 10^9^/L, hemoglobin 12.4 mg/dL, platelet 410 × 10^9^/L), liver function test (normal), and renal panel (normal). Thyroid function test, rheumatoid factor, and anti-cyclic citrullinated peptide were normal. As there was a suspicion of acromegaly, we investigated the levels of insulin-like growth factor-1 and performed an oral glucose tolerance test; the results of both of these tests were normal.

**Fig. 3:**
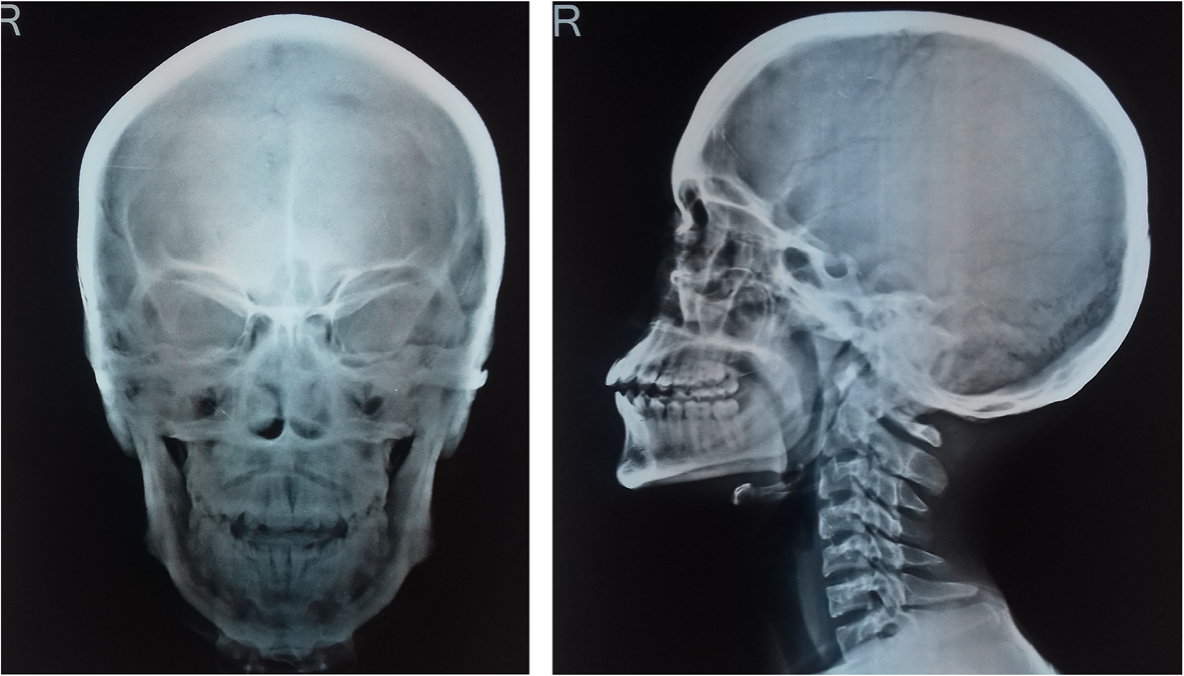
Radiography of skull showed mild cortical and subperiosteal thickening

**Fig. 4:**
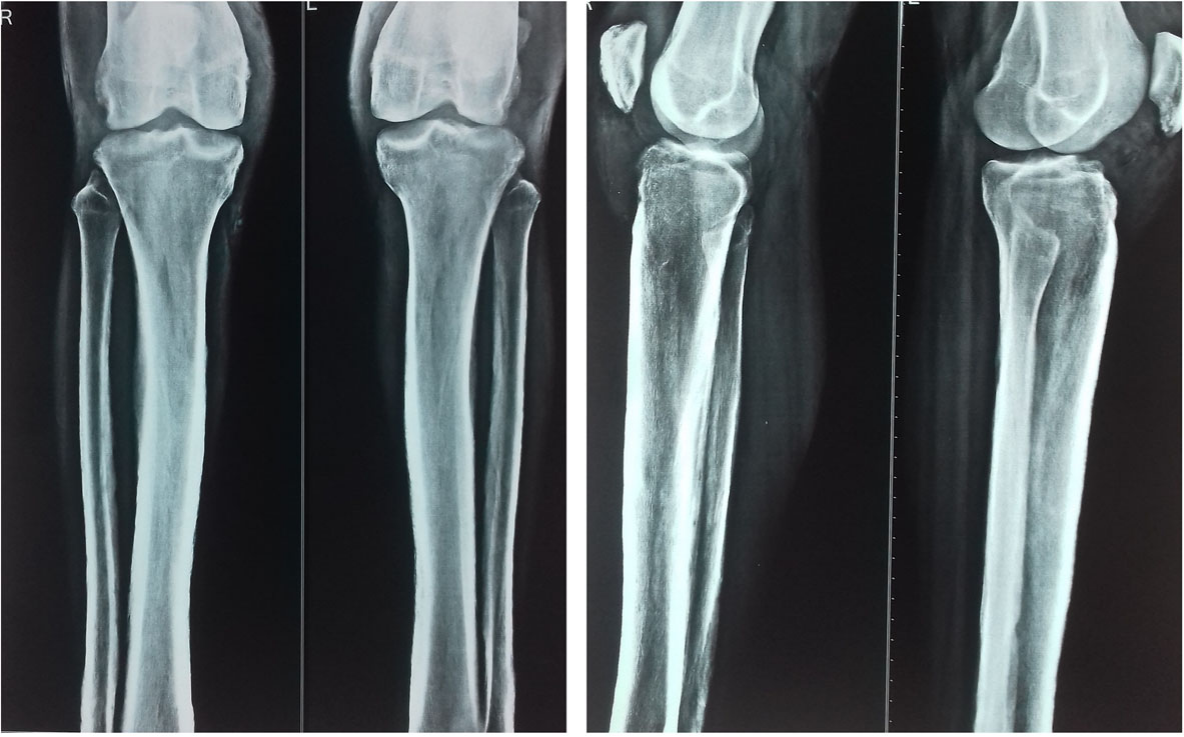
Radiograph of bilateral knees showing periosteal hypertrophy with subperiosteal bone formation

**Fig. 5:**
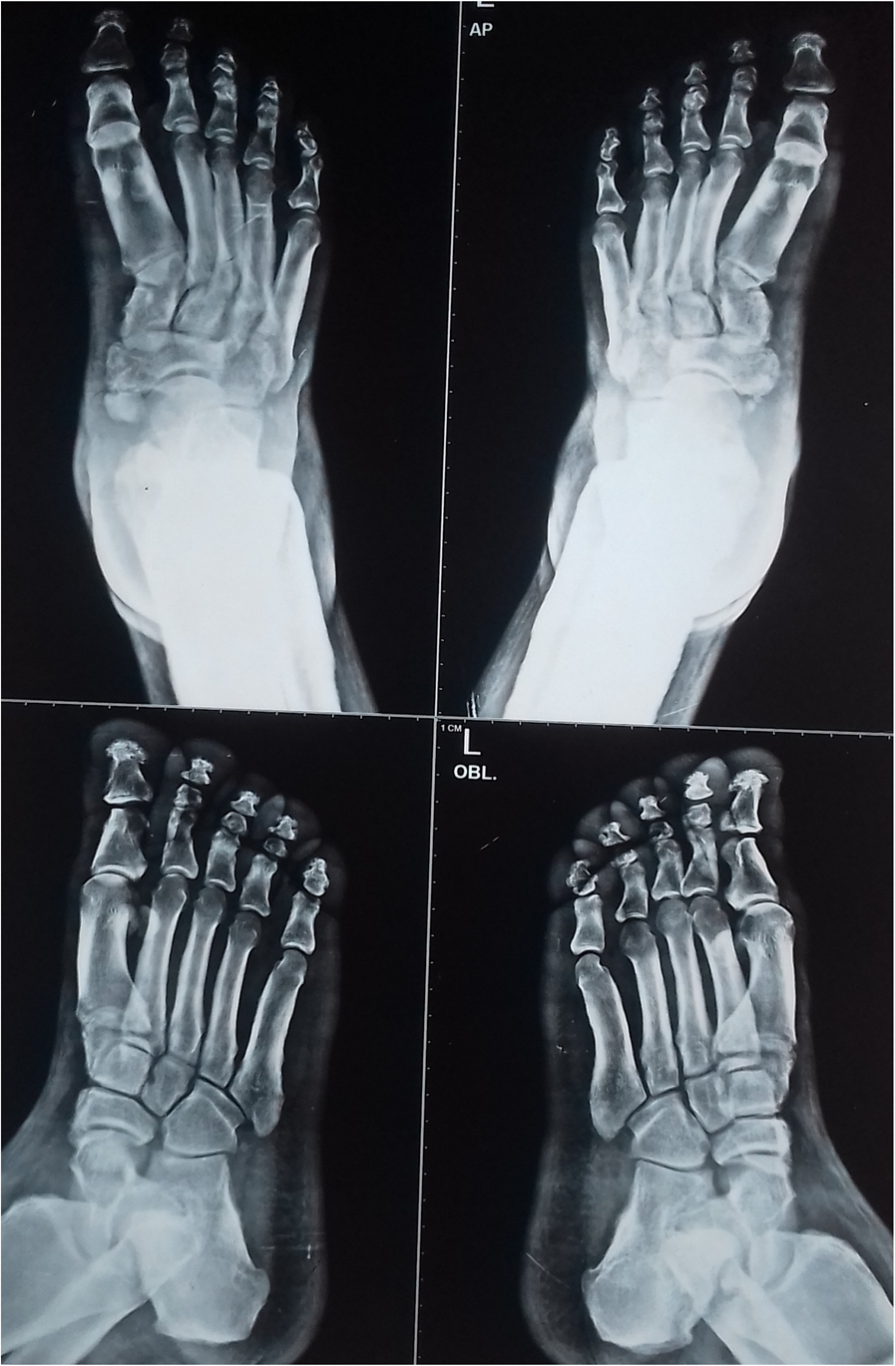
Radiograph of bilateral ankles showing irregular subperiosteal bone formation and cortical thickening of distal tibia, fibula, calcaneum, and talus

**Fig. 6:**
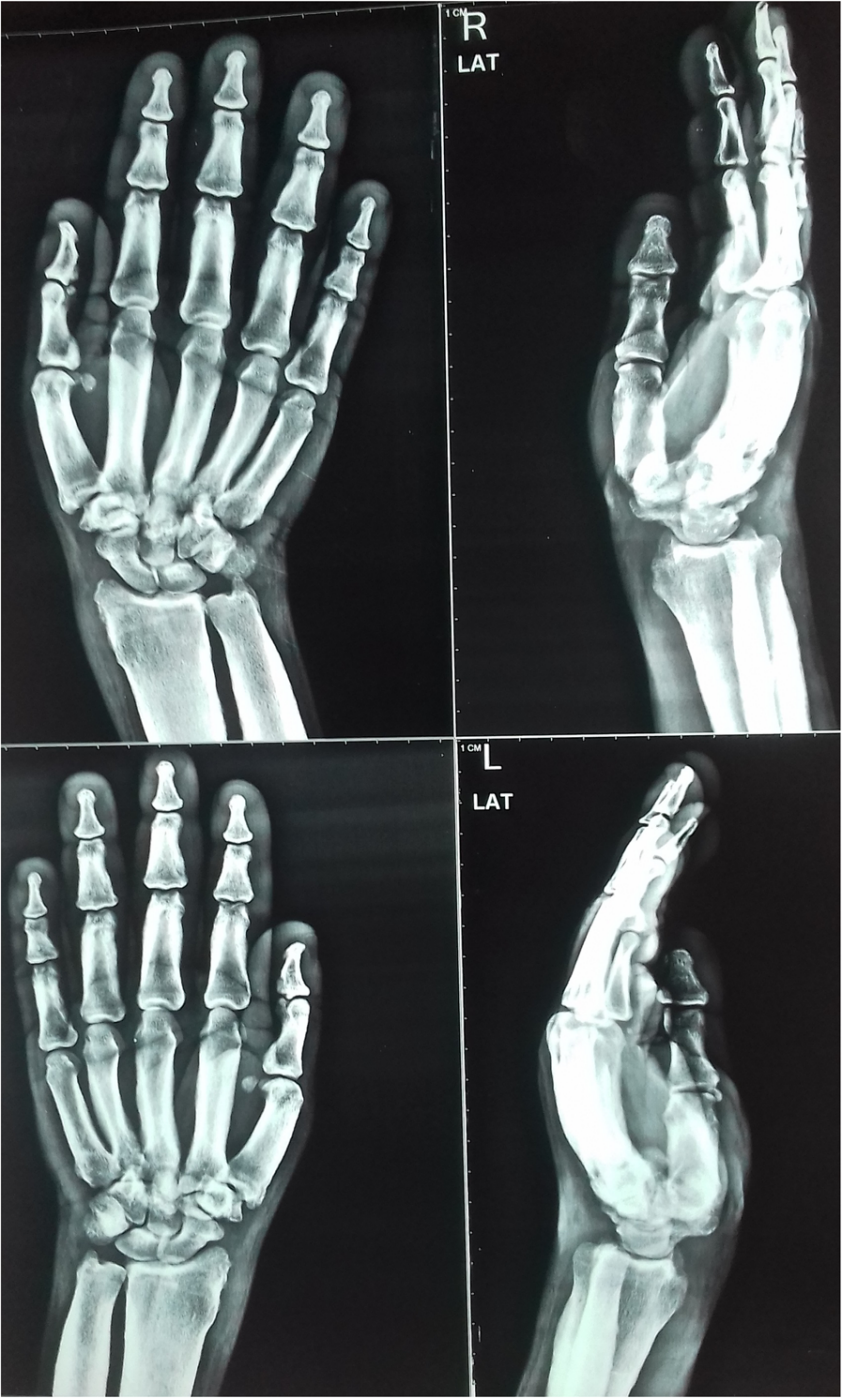
Radiograph of bilateral hands showing soft tissue tumefaction, hyperostosis of metacarpal, and proximal phalanges

Radiography of the skull showed mild cortical and subperiosteal thickening (Fig. 3). Bilateral knee x-rays revealed symmetrical, irregular, and periosteal hypertrophy with subperiosteal new bone formation in the proximal tibia and fibula and distal femur (Fig. 4). Radiographs of bilateral ankles demonstrated irregular subperiosteal new bone formation and cortical thickening of distal tibia, fibula, calcaneum, and talus (Fig. 5). The x-rays of bilateral hands showed soft tissue tumefaction, particularly in the distal phalanges and periostitis and hyperostosis of metacarpal and proximal phalanges (Fig. 6).

Aspiration of synovial fluid from knee joint was done to rule out other forms of arthritis. Synovial fluid analysis proved it to be non-inflammatory, non-hemorrhagic, and non-septic.

The patient stayed in our center for 6 days and was managed with selective COX-2 inhibitors (Eltrocoxib 60 mg PO, OD), steroids (Prednisolone 5 mg PO, OD), oral retinoids (20 mg morning and 10 mg HS), and retinoid ointment. The joint pain and swelling improved markedly with treatment. He was subsequently discharged with outpatient follow-up scheduled 1 and 6 months later, respectively.

However, the patient has since returned to his hometown and missed his physical appointments. Follow-up was established via telephone on both occasions, and it was found that his joint pain and swelling was minimal and the pachyderma had reduced gradually since discharge. The patient experienced no relapses or complications from the condition or the medications.

## Discussion

We reported a case of a 19-year-old patient with chief complaints of pain and progressive enlargement of hands and feet along with profuse sweating and gradual coarsening of facial features. Examination findings, laboratory investigations, and radiographic analyses all pointed towards a diagnosis of PDP.

Although the diagnosis of PDP can be made on the basis of classic clinical and radiological features, it is often overlooked due to the lack of qualified health workers and limited resources in the context of Nepal; to our knowledge, there is only one other known case report on this disease entity in the local region [15]. The case described here is that of a complete form of PDP, for which the key supportive clinical and radiological features and the relevant differential diagnoses, taken in consideration of the locoregional context, form the main points of discussion.

Hypertrophic osteoarthropathy (HOA) can be divided into primary and secondary forms. PDP, the primary form, accounts for 5% of all cases of HOA. Secondary HOA, also called pulmonary HOA, is associated with underlying cardiopulmonary diseases and malignancies [16].

PDP was first described by Friedreich in 1868, who called it “*hyperostosis of the entire skeleton*” [16]. In 1907, Unna coined the term ‘cutis verticis gyrate’ when describing the thick, transversely folded skin of the scalp and forehead seen in PDP patients [17].

The diagnostic criteria for PDP include major criteria consisting of pachyderma, periostosis, and finger clubbing, as well as minor criteria including hyperhidrosis, arthralgia, gastric ulcer, cutis verticis gyrate, blepharoptosis, joint effusion, column-like legs, edema, seborrhea, acne, and flushing [18, 19]. Our patient met all three major criteria and was diagnosed with a complete form of PDP.

This syndrome can be distinguished from acromegaly on the basis of clinical features and laboratory findings. In contrast to PDP, acromegaly presents clinically with larger bones in the face, skull and limbs, jaw prognathism, along with elevated insulin-like growth factor-1 levels and positive oral glucose tolerance test [20–22]. Acromegaly is often caused by a pituitary tumor, and the potential manifestations of the tumor’s local compression and hormonal disruption additionally help to distinguish it from PDP. In our patient, closer scrutiny in the clinical examination coupled with the negative biochemical markers for acromegaly effectively allowed us to rule out this differential.

The clinical presentation can also mimic certain types of psoriatic arthritis such as psoriatic onycho-pachydermo-periostitis [23]. Limited to the extremities and characterized by psoriatic nail involvement, psoriatic onycho-pachydermo-periostitis manifests as thickening of the soft tissues of the fingers and toes, osteoperiostitis of the distal phalanges, and psoriatic skin lesions [24]. Rheumatoid arthritis may also mimic the presentation with its joint features [24]. In our patient, the history was not supportive of either disease entity. Moreover, the erythrocyte sedimentation rate was normal and the joint aspirate revealed a synovial fluid that was non-inflammatory in nature.

Thyroid acropachy, a rare complication of autoimmune thyroid disease, should also be considered in this context. Characterized by progressive exophthalmos, relatively symmetric swelling of the hands and feet, clubbing of the digits, and pretibial myxedema, thyroid acropachy can manifest as periostosis and arthralgia, closely resembling PDP [25]. In our patient, clinical history and examination suggested the patient was euthyroid, and the subsequent thyroid function test was normal.

Other less common disease entities, such as fluorosis, hypervitaminosis A, and Caffey’s disease, are possible differentials in a patient presenting with signs and symptoms consistent with periostosis [19, 26, 27]. These were less likely given the lack of clinical and radiological features supportive of these diagnoses.

HOA is characterized by a symmetric periosteal reaction on radiographic examination. This periosteal reaction classically occurs in the long bones and also in the phalanges. In the long bones, the diaphysis is typically affected first, with involvement of the metaphysis and epiphysis indicating progression of disease [1]. Involvement of the epiphyseal region distinguishes it from the secondary form, in which the epiphyses are usually spared [28, 29]. There is typically no abnormality of the marrow or soft tissue adjacent to the periosteal reaction of HOA, and involvement of these tissues should suggest the possibility of other diagnoses [1]. Radiographs may also show acro-osteolysis or tuft hypertrophy of the phalanges [30]. In the joints, HOA classically produces soft tissue swelling without joint space narrowing, erosions, or other arthritic changes [1].

Radiographic findings in our case were similar to those reported by Sasane et al. [31], except for radiography of the skull, which was unremarkable in their case. In addition to the radiographic findings seen in our case, Rastogi et al. [28] also revealed spondylotic changes in spine, with loss of lumbar lordosis and new bone formation along the iliac bones. A recent case-series from Tunisia also revealed marked periostosis of the long bones in all cases [32]. In addition to knee findings seen in our case, the case reported by Guerini et al. [33] showed calcification at the insertion of the quadriceps tendon at the upper edge of the left patella. Our radiographic findings are similar to Reginato et al. [6], Matucci-Cerinic et al. [18], and Abdullah et al. [22].

Due to the central role of PGE2 in PDP pathogenesis, management of PDP is generally symptomatic using non-steroidal anti-inflammatory drugs, corticosteroids, or colchicine for pain relief [34]. Recent advances include trials of oral etoricoxib or bisphosphonates for arthritis coupled with arthroscopic synovectomy [35, 36].

## Conclusion

Clinical presentations of PDP can be confused with secondary hypertrophic osteoarthropathy, psoriatic arthritis, rheumatoid arthritis, thyroid acropachy, and acromegaly. PDP should be considered as differential diagnosis when a patient presents with HOA and acromegaloid features.

## Abbreviations

HOA: Hypertrophic osteoarthropathy
PDP: Pachydermoperiostosis
PGE2: Prostaglandin E2
